# Utility of [^68^Ga]FAPI-04 and [^18^F]FDG dual-tracer PET/CT in the initial evaluation of gastric cancer

**DOI:** 10.1007/s00330-022-09321-1

**Published:** 2022-12-16

**Authors:** Ying Miao, Runhua Feng, Rui Guo, Xinyun Huang, Wangxi Hai, Jian Li, Teng Yu, Qian Qu, Min Zhang, Chengfang Shangguan, Jun Mi, Zhenggang Zhu, Biao Li

**Affiliations:** 1 Department of Nuclear Medicine, Ruijin Hospital, Shanghai Jiao Tong University School of Medicine, 197 Ruijin Er Road, Shanghai, 200025 China; 2 Department of General Surgery, Ruijin Hospital, Shanghai Jiao Tong University School of Medicine, 197 Ruijin Er Road, Shanghai, 200025 China; 3 Clinical Research Center, Ruijin Hospital, Shanghai Jiao Tong University School of Medicine, 197 Ruijin Er Road, Shanghai, 200025 China; 4 Department of Pathology, Ruijin Hospital, Shanghai Jiao Tong University School of Medicine, 197 Ruijin Er Road, Shanghai, 200025 China; 5 Department of Oncology, Ruijin Hospital, Shanghai Jiao Tong University School of Medicine, 197 Ruijin Er Road, Shanghai, 200025 China; 6 Key Laboratory of Cell Differentiation and Apoptosis of Chinese Ministry of Education, Shanghai Jiao Tong University School of Medicine, 280 South Chongqing Road, Shanghai, 200025 China; 7 Collaborative Innovation Center for Molecular Imaging of Precision Medicine, Ruijin Center, 197 Ruijin Er Road, Shanghai, 200025 China

**Keywords:** Gastric cancer, [^68^Ga]FAPI-04, [^18^F]FDG, PET/CT

## Abstract

**Objectives:**

We aimed to investigate the role of [^68^Ga]FAPI-04 and [^18^F]FDG dual-tracer PET/CT for the initial assessment of gastric cancer and to explore the factors associated with their uptake.

**Methods:**

This study enrolled 62 patients with histopathologically confirmed gastric cancer. We compared the diagnostic performance of [^68^Ga]FAPI-04, [^18^F]FDG, and combined dual-tracer PET/CT. The standardized uptake value (SUV) and tumor-to-background ratio (TBR) were also measured, and the factors that influence tracer uptake were analyzed.

**Results:**

[^68^Ga]FAPI-04 PET/CT detected more primary lesions (90.3% vs 77.4%, *p* = 0.008) and peritoneal metastases (91.7% vs 41.7%, *p* = 0.031) and demonstrated higher SUV_max_ and TBR values (*p* < 0.001) of primary lesions compared to [^18^F]FDG PET/CT. Dual-tracer PET/CT significantly improved the diagnostic sensitivity for the detection of distant metastases, compared with stand-alone [^18^F]FDG (97.1% vs 73.5%, *p* = 0.008) or [^68^Ga]FAPI-04 (97.1% vs 76.5%, *p* = 0.016) PET/CT. Subsequently, treatment strategies were changed in nine patients following [^68^Ga]FAPI-04 and [^18^F]FDG dual-tracer PET/CT. Nevertheless, [^68^Ga]FAPI-04 uptake was primarily influenced by the size and invasion depth of the tumor. Both [^68^Ga]FAPI-04 and [^18^F]FDG PET/CT showed limited sensitivity for detecting early gastric cancer (EGC) (37.5% vs 25.0%, *p* > 0.05).

**Conclusions:**

In this initial study, [^68^Ga]FAPI-04 and [^18^F]FDG dual-tracer PET/CT were complementary and improved sensitivity for the detection of distant metastases pre-treatment in gastric cancer and could improve treatment stratification in the future. [^68^Ga]FAPI-04 had limited efficacy in detecting EGC.

**Key Points:**

*•* *[*^*68*^*Ga]FAPI-04 and* *[*^*18*^*F]FDG dual-tracer PET/CT are complementary to each other for improving diagnostic sensitivity in the initial evaluation of distant metastases from gastric cancer.*

*•* *[*^*68*^*Ga]FAPI-04 PET/CT showed limited sensitivity in detecting EGC.*

*• Need for further validation in a larger multi-centre prospective study.*

**Supplementary Information:**

The online version contains supplementary material available at 10.1007/s00330-022-09321-1.

## Introduction

Gastric cancer ranks as the fifth and fourth in cancer incidence and cancer-related deaths globally, respectively [1]. Patients are frequently diagnosed with advanced gastric cancer (AGC) due to the insidious early symptoms. Treatment of gastric cancer is currently based on multidisciplinary management, including surgery, systemic chemotherapy, radiotherapy, immunotherapy, and targeted therapy [2]. Accurate evaluation of disease extent is paramount for selecting the appropriate treatment method. [^18^F]FDG PET/CT imaging for gastric cancer can sometimes be suboptimal, particularly in individuals with non-intestinal-type gastric cancers or individuals with signet ring cell carcinomas (SRCC) or mucinous adenocarcinomas (MAC) [3, 4].

Fibroblast activation protein (FAP) is commonly overexpressed in cancer-associated fibroblasts, which are known to be the primary components of stromal cells that contribute up to 90% of the tumor mass [5, 6]. Recently, ^68^Ga-labeled quinoline-based FAP inhibitor (FAPI) has allowed for the imaging of tumor stroma by targeting FAP, among which [^68^Ga]FAPI-04 has exhibited favorable tumor-to-background ratio (TBR) and kinetics [7, 8]. [^68^Ga]FAPI-04 PET/CT reportedly outperformed [^18^F]FDG PET/CT, especially in cancers of unknown primary origin, breast cancer, and several digestive system tumors, including gastric cancer; thus, it may be an alternative to [^18^F]FDG PET/CT in the detection of these tumors [9, 10]. However, the number of SRCC patients enrolled in previous studies on gastric cancer was limited. Additionally, elevated FAP expression has also been observed during wound healing and matrix remodeling, including chronic inflammation, atherosclerosis, and liver and lung fibrosis [6]. Whether [^68^Ga]FAPI-04 PET/CT could replace or supplement [^18^F]FDG PET/CT in the initial evaluation of gastric cancer needs to be further investigated.

Based on the comparison of [^68^Ga]FAPI-04 and [^18^F]FDG PET/CT in a larger cohort, our research further explored the role of combined dual-tracer PET/CT in the initial assessment of gastric cancer and analyzed the clinicopathological factors that influence tracer uptake.

## Material and methods

### Patients

**Fig. 1:**
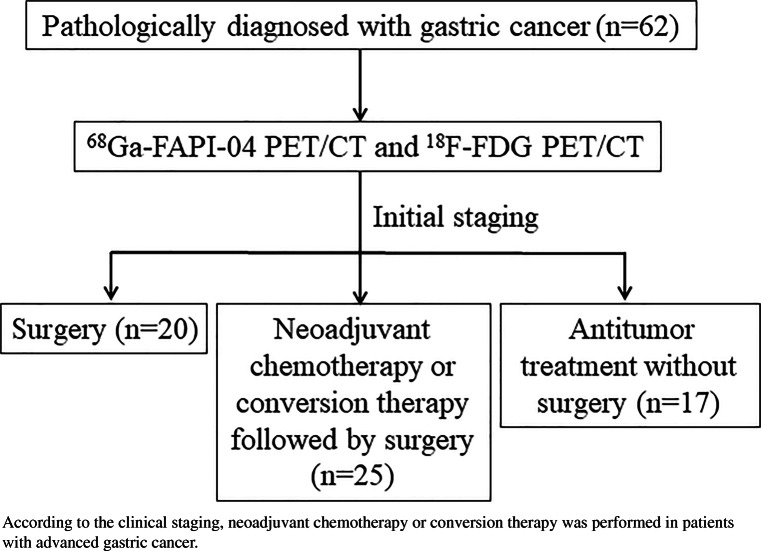
Study flowchart

The Ruijin Hospital Ethics Committee of Shanghai Jiao Tong University School of Medicine approved this prospective clinical study (2020 CER No.172). This study enrolled 62 patients pathologically diagnosed with gastric cancer by gastroscopy biopsy for initial staging. All patients signed written informed consent prior to PET/CT imaging. Subsequently, [^68^Ga]FAPI-04 PET/CT and [^18^F]FDG PET/CT imaging were carried out before treatment. Following comprehensive imaging results, clinical evaluations, and patients’ willingness, 20 patients underwent primary surgery, 25 patients underwent chemotherapy followed by surgery (including 19 patients who received neoadjuvant chemotherapy and 6 patients who received conversion therapy), and 17 patients underwent antitumor treatment without surgery. Figure 1 shows the study flowchart. Table 1 summarizes the clinicopathological characteristics of the 62 patients. TNM staging was classified according to the eighth edition of the American Joint Committee on Cancer TNM system.

**Table 1:** Clinicopathological characteristics of patients

Characteristic	Number	%
No. of patients	62	
Age (years)		
Median	64	
Range	24–75	
Sex		
Male	44	71.0
Female	18	29.0
Histologic type		
PCC	27	43.5
non-PCC	35	56.5
Pathological tumor staging		
pT1	8	12.9
pT2–T4a	12	19.4
ypT0–T4a	25	40.3
N/A	17	27.4
Pathological lymph node staging		
pN0	9	14.5
pN1–N2	11	17.8
ypN0–N3a	25	40.3
N/A	17	27.4
Degree of differentiation		
Well	0	0.0
Moderately	16	25.8
Poorly	34	54.8
N/A	12	19.4
Lauren classification		
Intestinal subtype	20	32.3
Non-intestinal subtype	16	25.8
N/A	26	41.9

*PCC* poorly cohesive carcinoma (including signet ring cell carcinoma), *N/A* not applicable, *ypTNM* The 8th American Joint Committee on Cancer Post Neoadjuvant Therapy Classification staging system

Histological type, degree of differentiation, and Lauren classification were based on known gastroscopy biopsy or postoperative pathology results

### Radiopharmaceuticals

[^68^Ga]FAPI-04 was prepared following the prior approach [7]. Briefly, radioactive gallium (^68^Ga) was eluted from a ^68^Ge-/^68^Ga generator and added to a reactor vial containing 20 ug of DOTA-FAPI-04 (CSBio), then mixed with NaOAc (1 mol/L, 1 mL), which resulted in a pH of 4. The mixture was further reacted at 100°C for 10 minutes using an automatic synthesis module (Trasis). [^18^F]FDG was synthesized routinely. The products were purified with radiochemical purity > 95% prior to clinical use. Both [^68^Ga]FAPI-04 and [^18^F]FDG were prepared in the Radiochemistry Facility of PET/CT Center, Ruijin Hospital.

### PET/CT imaging

Both [^68^Ga]FAPI-04 PET/CT and [^18^F]FDG PET/CT were performed on a specialized PET/CT scanner (Biograph Vision 450, Siemens Healthineers). Whole-body PET/CT (from the top of the head to the upper thigh or from the base of the head to the upper thigh with the head scanned separately) was carried out 30–60 min after injection of 1.85–2.96 MBq of [^68^Ga]FAPI-04 per kilogram of body weight (kg/bw) and 60–90 min after injection of 3.7–4.44 Mbq of [^18^F]FDG/kg/bw. After excluding drug contraindications, 20 mg of hyoscine butylbromide was injected intravenously before scanning, followed by drinking approximately 500 mL of water to achieve gastric distension [11, 12]. Diagnostic non-contrast-enhanced CT (non-CECT) scans were performed using the CARE Dose 4D technique (120 kV, automatic mA-modulation). PET images were obtained in 3D mode and reconstructed in a 440 × 440 matrix size (iteration: 4, subset: 5) using the TrueX + TOF (ultraHD-PET) method. The interval between the two PET/CT scans was within 9 days.

### Image analysis

Two experienced nuclear medicine physicians (G.R. and H.X.Y., with 12 and 5 years of experience in nuclear oncology, respectively) independently analyzed the [^68^Ga]FAPI-04 PET/CT and [^18^F]FDG PET/CT images. A positive dual-tracer PET/CT was defined as [^68^Ga]FAPI-04 PET/CT-positive or [^18^F]FDG PET/CT-positive. For semiquantitative analysis, a spherical region of interest was delineated around the tumor lesions, which was automatically adjusted to a 3D volume of interest (VOI) at a 60% isocontour using syngo.via software (Siemens Healthineers), and the maximum standardized uptake value (SUV_max_) was recorded. Additionally, a 10-mm diameter VOI was placed over the non-lesional gastric wall to obtain the SUV_max_ of the normal gastric wall background, a 10-mm diameter VOI was drawn on the descending aorta to acquire the mean standardized uptake value (SUV_mean_) of the mediastinal blood pool background, and a 20-mm diameter VOI was set on the non-lesional right liver lobe to obtain the SUV_mean_ of liver blood pool background [13]. The TBR was displayed as TBR-G, TBR-A, and TBR-L, which were calculated by dividing the SUV_max_ of the gastric tumors with the background of the normal gastric wall, mediastinal blood pool, and liver blood pool, respectively. Histopathological findings, laparoscopic exploration, and contemporaneous and follow-up imaging were the reference standards for the final diagnosis. Progression of metastatic lesions or reduction in the size/number of lesions after chemotherapy on follow-up imaging was considered a malignant feature [14].

### Statistical analysis

IBM SPSS Statistics 26.0 was used for statistical analysis. Continuous variables were presented as medians and interquartile range (IQR), whereas categorical variables were presented as numbers and percentages. The diagnostic performance, including sensitivity, specificity, accuracy, positive predictive value, and negative predictive value, was analyzed. The comparison of SUV_max_ or TBR between [^68^Ga]FAPI-04 and [^18^F]FDG PET/CT was conducted using the Wilcoxon signed-rank test. The Mann–Whitney U test was used to compare SUV_max_ within groups. The comparison of diagnostic performance between and within groups was performed using the McNemar test, *χ*^2^ test, or Fisher’s exact test. All statistical tests were two-sided, and a value of *p* < 0.05 was considered statistically significant.

## Results

### Performance of [^68^Ga]FAPI-04, [^18^F]FDG, and dual-tracer PET/CT in diagnosing primary lesions

**Fig. 2:**
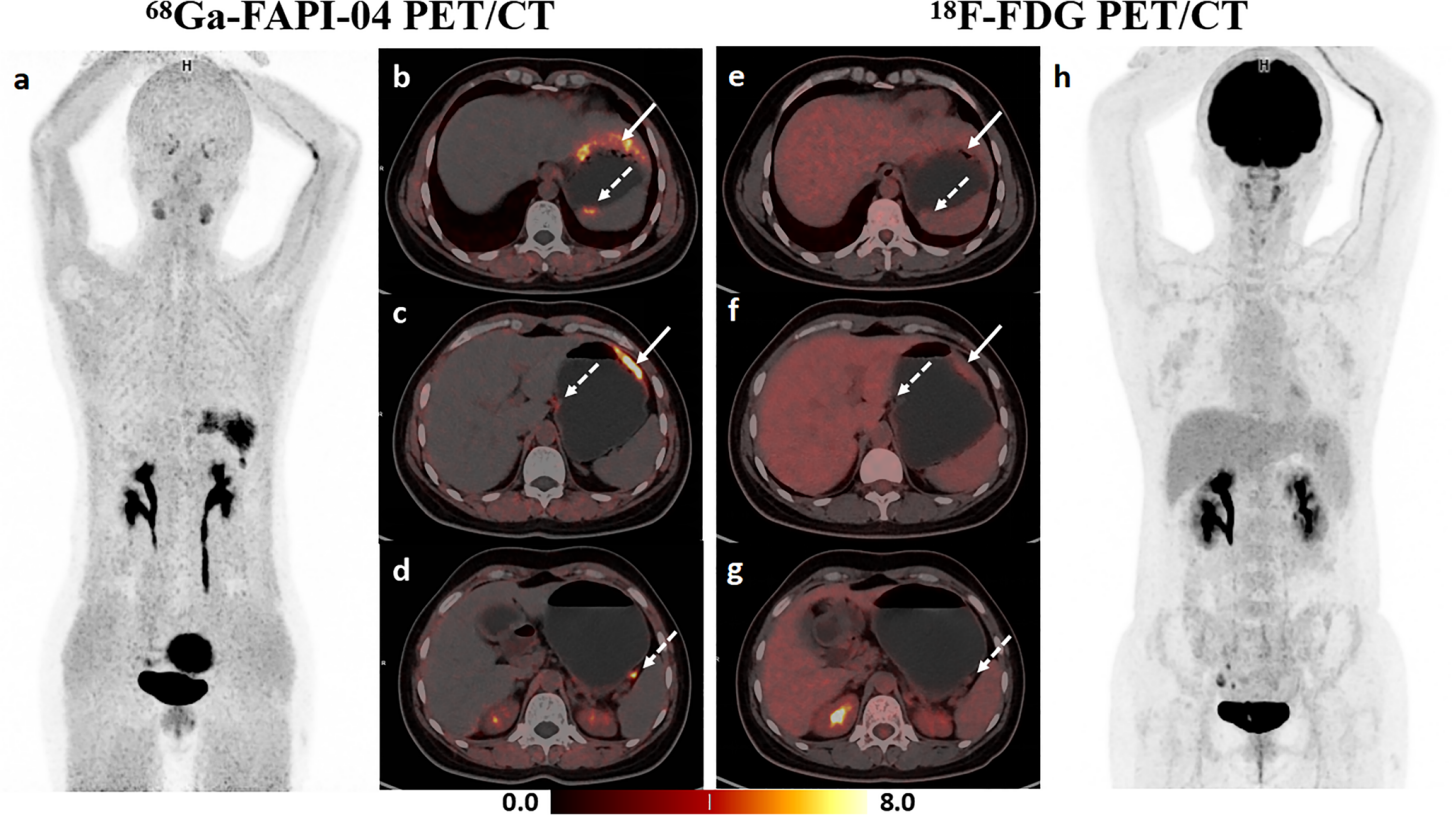
A 44-year-old female patient was histopathologically diagnosed with poorly cohesive carcinoma (with partial signet ring cell carcinoma) in the greater curvature of the gastric body and posterior wall of the gastric fundus and had perigastric lymph node metastases. **a–d** [^68^Ga]FAPI-04 PET/CT imaging. Maximal intensity projection (MIP) image of [^68^Ga]FAPI-04 PET (**a**), clear identification of gastric cancer lesions (solid arrow in **b**, **c** and dotted arrow in **b**) and perigastric metastatic lymph nodes (dotted arrow in **c**, **d**). **e–h** [^18^F]FDG PET/CT imaging. MIP image of [^18^F]FDG PET (**h**), the gastric lesion in the greater curvature of the gastric body (solid arrow in **e**, **f**) displayed diffuse mild uptake, the lesions in the posterior wall of the gastric fundus (dotted arrow in **e**) and perigastric metastatic lymph nodes (dotted arrow in **f**, **g**) showed negative uptake

Table 2 summarizes the sensitivity of [^68^Ga]FAPI-04, [^18^F]FDG, and dual-tracer PET/CT in detecting primary lesions. In the overall cohort, [^68^Ga]FAPI-04 PET/CT detected more primary lesions compared to [^18^F]FDG PET/CT (56/62, 90.3% vs 48/62, 77.4%, *p* = 0.008). Furthermore, eight (12.9%) [^18^F]FDG PET/CT-negative patients detected by [^68^Ga]FAPI-04 PET/CT were confirmed as having poorly cohesive carcinoma (PCC), including SRCC. A representative case is indicated in Fig. 2. Six (9.7%) patients were both tracer-negative; four (66.7%) had SRCC. According to the depth of tumor invasion, the overall cohort was further divided into early gastric cancer (EGC) and AGC groups. [^68^Ga]FAPI-04 PET/CT showed superior sensitivity to [^18^F]FDG (53/54, 98.1% vs 46/54, 85.2%, *p* = 0.016) in detecting AGC, whereas no statistical difference was noted between the two in detecting EGC (3/8, 37.5% vs 2/8, 25.0%, *p* > 0.05). The sensitivity of dual-tracer PET/CT was equivalent to that of [^68^Ga]FAPI-04 (*p* > 0.05) and superior to that of [^18^F]FDG in both the overall cohort (*p* = 0.008) and in detecting AGC (*p* = 0.016). Furthermore, both [^68^Ga]FAPI-04 and dual-tracer PET/CT were more sensitive in detecting AGC than EGC (53/54, 98.1% vs 3/8, 37.5%, *p* < 0.001), and the results were similar for [^18^F]FDG (46/54, 85.2% vs 2/8, 25.0%, *p* = 0.001).

**Table 2:** Sensitivity of ^68^Ga-FAPI-04, ^18^F-FDG, and dual-tracer PET/CT in detecting primary lesions of gastric cancer

Group	No. of patients	^68^Ga-FAPI-04		^18^F-FDG		Dual-tracer	
		*N*	%	*N*	%	*N*	%
Total	62	56/62	90.3	48/62	77.4	56/62	90.3
EGC	8	3/8	37.5	2/8	25.0	3/8	37.5
AGC	54	53/54	98.1	46/54	85.2	53/54	98.1

*EGC* early gastric cancer, *AGC* advanced gastric cancer*, Dual-tracer* FAPI (+)-FDG (+) or FAPI (+)-FDG (**−**) or FAPI (**−**)-FDG (+)

Biopsy pathologies confirmed gastric cancer in 62 patients. Early gastric cancer and advanced gastric cancer were determined based on pathological and clinical staging

### Performance of [^68^Ga]FAPI-04, [^18^F]FDG, and dual-tracer PET/CT in diagnosing regional lymph node metastases

Table 3 summarizes the performance of [^68^Ga]FAPI-04, [^18^F]FDG, and dual-tracer PET/CT in diagnosing regional lymph node metastases. A patient-based analysis was conducted in 20 patients who underwent surgery without preoperative antitumor treatment. Of these, 11 (55.0%) were pathologically confirmed as having regional nodal metastases. [^68^Ga]FAPI-04 and [^18^F]FDG PET/CT missed to detect nodal metastases in four (36.4%) and five (45.5%) patients, respectively, whereas the false-negative patients missed by each were slightly different. In detecting regional nodal metastases, the sensitivity, specificity, and accuracy of [^68^Ga]FAPI-04 PET/CT were not significantly higher than those of [^18^F]FDG (*p* > 0.05). Additionally, dual-tracer PET/CT revealed comparable performance in diagnosing regional nodal metastases compared with either single-tracer PET/CT (*p* > 0.05).

**Table 3:** Performance of [^68^Ga]FAPI-04, [^18^F]FDG, and dual-tracer PET/CT in diagnosing regional nodal metastases of gastric cancer

Diagnostic performance	[^68^Ga]FAPI-04		[^18^F]FDG		Dual-tracer	
	*N*	%	*N*	%	*N*	%
Sensitivity	7/11	63.6	6/11	54.5	8/11	72.7
Specificity	8/9	88.9	7/9	77.8	7/9	77.8
Accuracy	15/20	75.0	13/20	65.0	15/20	75.0
PPV	7/8	87.5	6/8	75.0	8/10	80.0
NPV	8/12	66.7	7/12	58.3	7/10	70.0

*PPV* positive predictive value, *NPV* negative predictive value, *Dual-tracer* FAPI (+)-FDG (+) or FAPI (+)-FDG (**−**) or FAPI (**−**)-FDG (+)

The lymph node analysis was based on pathological results from 20 patients who underwent surgery without preoperative antitumor therapy

### Performance of [^68^Ga]FAPI-04, [^18^F]FDG, and dual-tracer PET/CT in diagnosing distant metastases

**Fig. 3:**
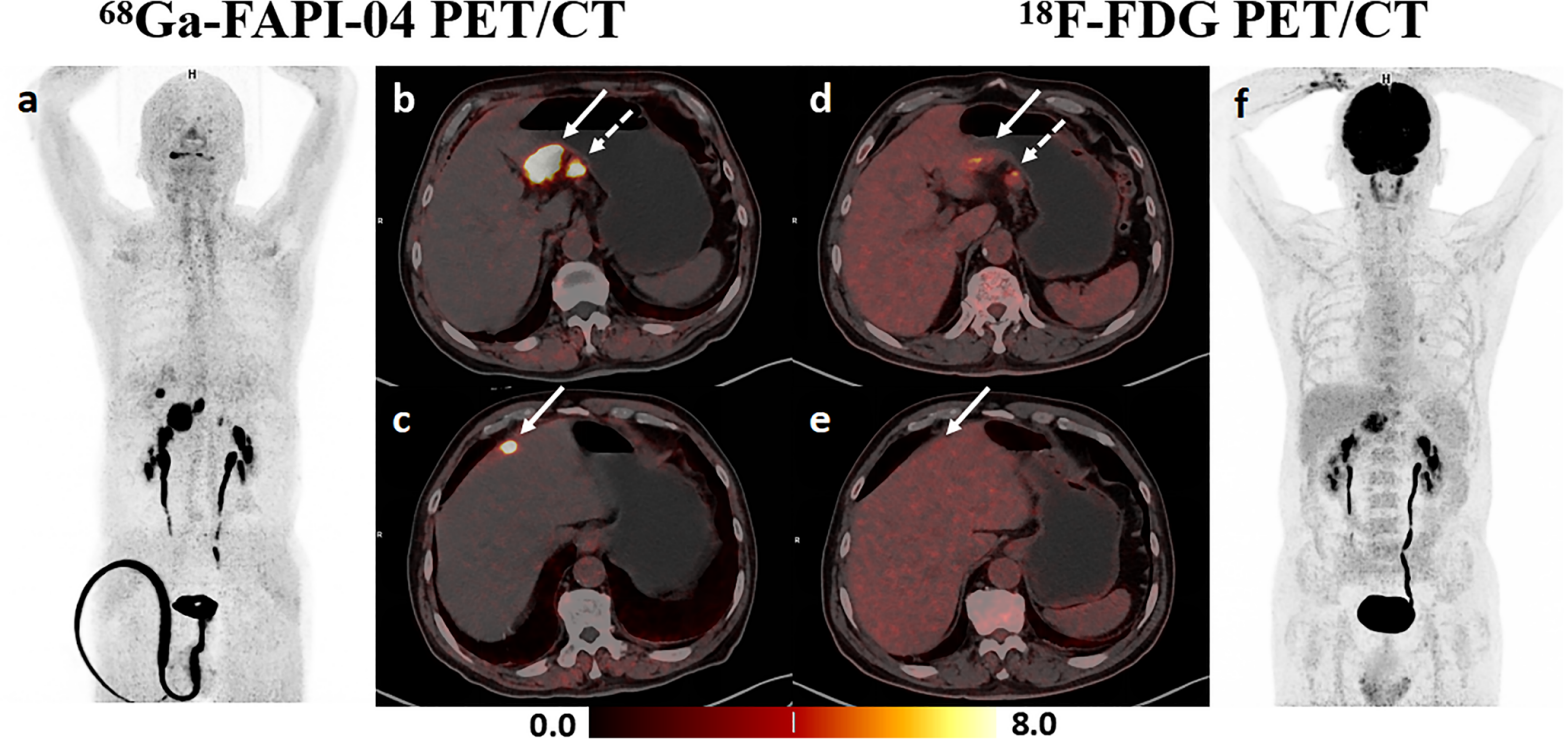
A 66-year-old male patient was histopathologically diagnosed with gastric antrum adenocarcinoma with perigastric lymph node metastases, and a false-positive uptake of [^68^Ga]FAPI-04 in the liver was proven to be a fibrotic nodule with calcified schistosome egg deposition without heterotypic findings. **a–c** [^68^Ga]FAPI-04 PET/CT imaging. Maximal intensity projection (MIP) image of [^68^Ga]FAPI-04 PET (**a**), clear recognition of gastric cancer lesion (solid arrow in **b**) and metastatic lymph node (dotted arrow in **b**), fibrotic nodule with calcified schistosome egg deposition mimicking liver metastasis (**c**). **d–f** [^18^F]FDG PET/CT imaging. MIP image of [^18^F]FDG PET (**f**), lower [^18^F]FDG uptake in gastric cancer lesion (solid arrow in **d**) and the metastatic lymph node (dotted arrow in **d**) compared with ^68^Ga-FAPI, liver lesion showed negative uptake (**e**)

**Fig. 4:**
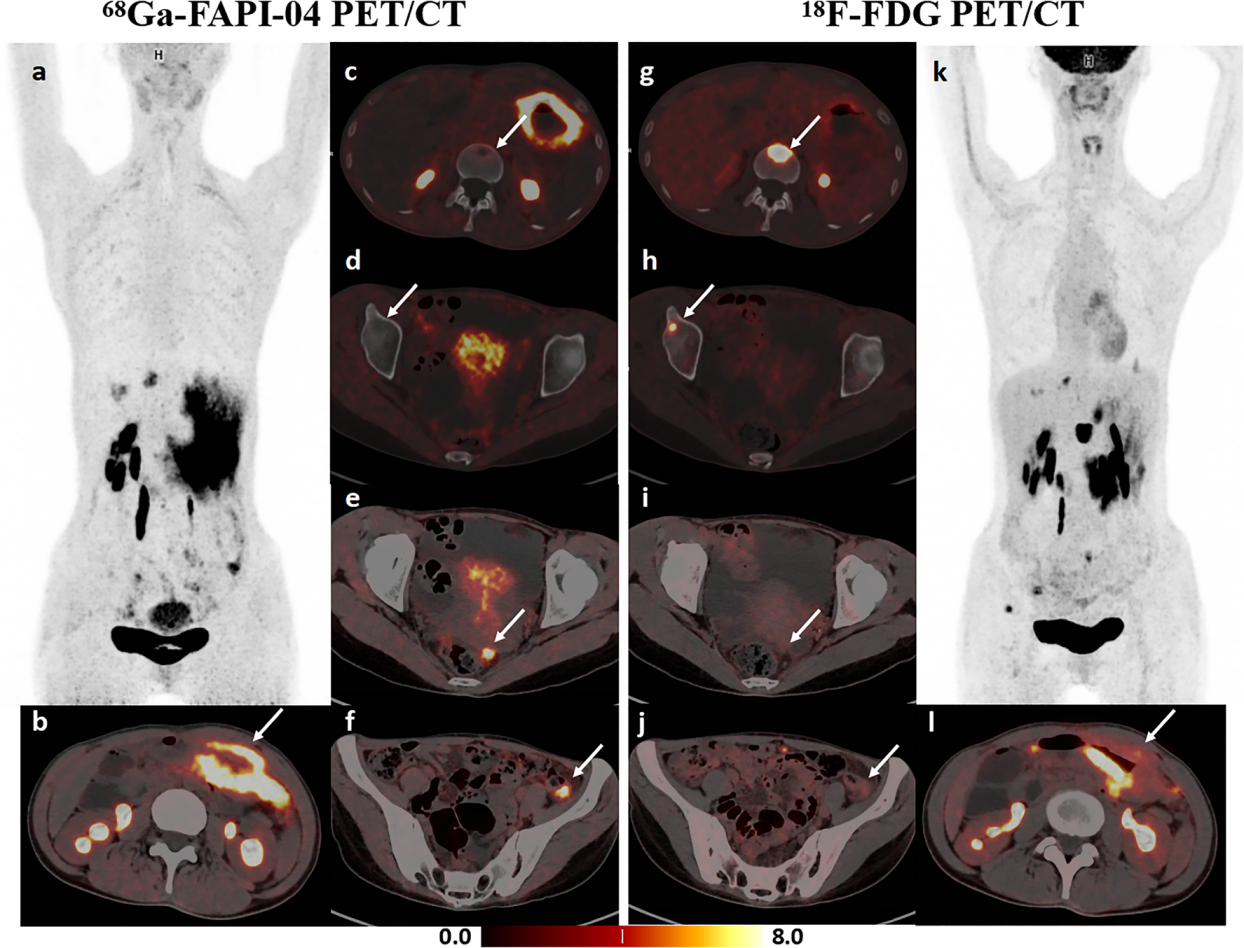
A 33-year-old female patient was histopathologically diagnosed with gastric adenocarcinoma (with partial signet ring cell carcinoma) with multiple metastatic nodules in the abdominopelvic cavity and peritoneum confirmed by laparoscopic exploration and bone metastases confirmed by follow-up imaging. **a–f** [^68^Ga]FAPI-04 PET/CT imaging. Maximal intensity projection (MIP) image of [^68^Ga]FAPI-04 PET (**a**), clear evidence of gastric cancer lesion (**b**) and peritoneal metastases (**e**, **f**), faint uptake in the L2 lumbar vertebra with a low focal density (**c**), no abnormal uptake in the right ilium (**d**). **g–l** [^18^F]FDG PET/CT imaging. MIP image of [^18^F]FDG PET (**k**), intense heterogeneous uptake in the gastric body lesion (**l**), strong support of bone metastases (**g**, **h**), false-negative uptake in peritoneal metastases (**i**, **j**)

Distant metastases were confirmed in 24 (38.7%) of the 62 patients. The sites of distant metastases included distant lymph nodes in 8 patients, peritoneum in 12, ovaries in 2, liver in 7, lung in 2, and bones in 3. Supplementary Table 1 lists the specific method for confirming distant metastases of gastric cancer. Table 4 summarizes the performance of [^68^Ga]FAPI-04, [^18^F]FDG, and dual-tracer PET/CT in diagnosing distant metastases. First, a patient-based analysis was conducted to compare the sensitivity of [^68^Ga]FAPI-04, [^18^F]FDG, and dual-tracer PET/CT in detecting distant metastases of gastric cancer to different sites. The sensitivity of [^68^Ga]FAPI-04 PET/CT in detecting peritoneal metastases was higher than that of [^18^F]FDG (11/12, 91.7% vs 5/12, 41.7%, *p* = 0.031). Second, a site-based analysis (based on the six categories of sites listed above, i.e., 328 sites, including 62 cases of distant lymph nodes, peritoneum, liver, lung and bones, and 18 cases of female ovaries) was performed to compare the performance of [^68^Ga]FAPI-04, [^18^F]FDG, and dual-tracer PET/CT in diagnosing distant metastases. Distant metastasis was confirmed in 34 of 328 sites (10.4%) in 62 patients following the reference standards. Both tracers missed a small peritoneal metastasis at the top of the diaphragm in one patient, which was diagnosed by laparoscopy and biopsy. Additionally [^18^F]FDG PET/CT missed seven peritoneal metastases, one ovarian metastasis, and one liver metastasis, and misinterpreted one distant nodal metastasis and one ovarian metastasis. In contrast, [^68^Ga]FAPI-04 PET/CT missed one distant nodal metastasis, one peritoneal metastasis, three liver metastases, two lung metastases, and one bone metastasis and misinterpreted one liver metastasis and one bone metastasis. Figure 3 depicts a false-positive uptake of [^68^Ga]FAPI-04 in a liver nodule. In detecting the overall distant metastases, [^68^Ga]FAPI-04 PET/CT demonstrated comparable sensitivity, specificity, and accuracy to [^18^F]FDG (*p* > 0.05). Meanwhile, the dual-tracer PET/CT sensitivity for detecting distant metastases was significantly higher compared with stand-alone [^68^Ga]FAPI-04 (*p* = 0.016) or [^18^F]FDG (*p* = 0.008) PET/CT; a typical case is indicated in Fig. 4. There was no statistical difference between the dual-tracer and either of the two single-tracer PET/CT for specificity and accuracy.

**Table 4:** Performance of [^68^Ga]FAPI-04, [^18^F]FDG, and dual-tracer PET/CT in diagnosing distant metastases of gastric cancer

	[^68^Ga]FAPI-04		[^18^F]FDG		Dual-tracer	
	*N*	%	*N*	%	*N*	%
Patient-based analysis of the diagnostic sensitivity						
Distant lymph nodes	7/8	87.5%	8/8	100.0%	8/8	100.0%
Peritoneum	11/12	91.7%	5/12	41.7%	11/12	91.7%
Ovaries	2/2	100.0%	1/2	50.0%	2/2	100.0%
Liver	4/7	57.1%	6/7	85.7%	7/7	100.0%
Lung	0/2	0.0%	2/2	100.0%	2/2	100.0%
Bones	2/3	66.7%	3/3	100.0%	3/3	100.0%
Site-based analysis of the diagnostic performance						
Sensitivity	26/34	76.5	25/34	73.5	33/34	97.1
Specificity	292/294	99.3	292/294	99.3	290/294	98.6
Accuracy	318/328	97.0	317/328	96.6	323/328	98.5
PPV	26/28	92.9	25/27	92.6	33/37	89.2
NPV	292/300	97.3	292/301	97.0	290/291	99.7

*PPV* positive predictive value, *NPV* negative predictive value, *dual-tracer* FAPI (+)-FDG (+) or FAPI (+)-FDG (**−**) or FAPI (**−**)-FDG (+)

The site-based analysis of the diagnostic performance was based on six categories of sites listed above (i.e., 328 sites, including 62 cases of distant lymph nodes, peritoneum, liver, lung and bones, and 18 cases of female ovaries)

### Comparison of [^68^Ga]FAPI-04 and [^18^F]FDG uptake and related clinicopathological factors

**Fig. 5:**
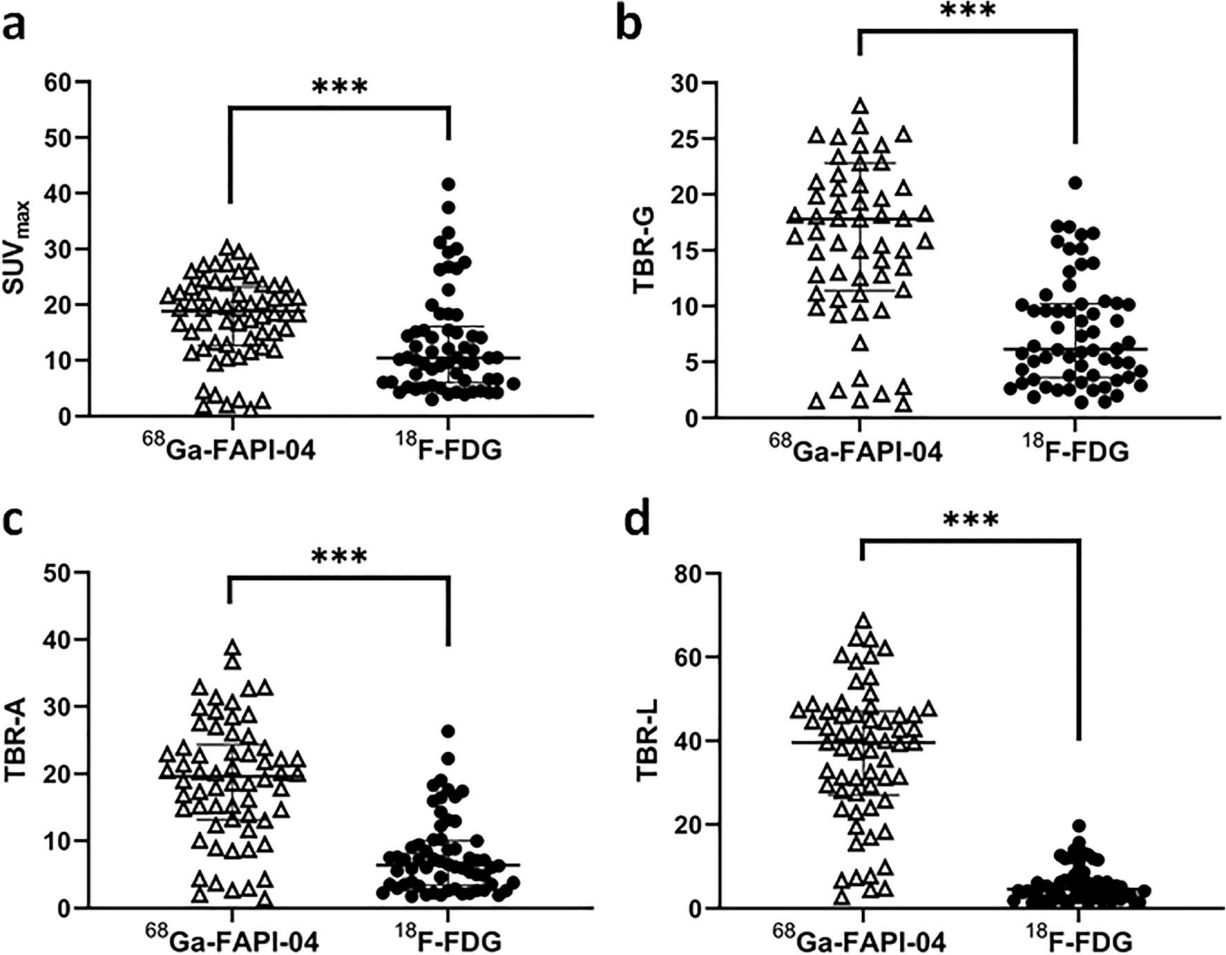
Comparison of [^68^Ga]FAPI-04 and [^18^F]FDG uptake in primary gastric tumors. **a**. Tumor SUV_max_ **b**. TBR-G: Tumor SUV_max_/normal gastric wall background SUV_max_ ratio **c**. TBR-A: Tumor SUV_max_/descending aorta background SUV_mean_ ratio **d**. TBR-L: Tumor SUV_max_/liver background SUV_mean_ ratio

[^68^Ga]FAPI-04 and [^18^F]FDG uptakes in primary gastric tumors are presented as SUV_max_, TBR-G, TBR-A, and TBR-L (Fig. 5). The median SUV_max_ of [^68^Ga]FAPI-04 was remarkably higher than that of [^18^F]FDG (18.81 vs 10.44, *p* < 0.001). The results were consistent with TBR when the normal gastric wall/descending aorta/liver backgrounds were subtracted. However, [^68^Ga]FAPI-04 and [^18^F]FDG uptake showed a more significant discrepancy in the TBR parameters, particularly TBR-L, which allows for visualizing gastric lesions adjacent to the liver.

Subgroup analysis was further performed to investigate the related clinicopathological factors that may affect [^68^Ga]FAPI-04 and [^18^F]FDG uptake. Table 5 shows the respective results. Both the median SUV_max_ of [^68^Ga]FAPI-04 and that of [^18^F]FDG were markedly higher in AGC compared to EGC and were also higher in tumors > 3 cm than in tumors ≤ 3 cm. Additionally, the median SUV_max_ of [^18^F]FDG was evidently lower in the subgroup of PCC (including SRCC) than that of non-PCC and was also lower in the subgroup of the non-intestinal type than that of the intestinal type. In contrast, the median SUV_max_ of [^68^Ga]FAPI-04 did not differ significantly between the subgroups according to histological type, Lauren classification, or degree of differentiation.

**Table 5:** Clinicopathological factors associated with [^68^Ga]FAPI-04 and [^18^F]FDG uptake in gastric cancer

Characteristic	N	[^68^Ga]FAPI-04			[^18^F]FDG		
		SUV_max_ median	SUV_max_ IQR	*p*	SUV_max_ median	SUV_max_ IQR	*p*
All	62	18.81	12.66, 23.18		10.44	5.97, 16.09	
Stage	62			< 0.001^***^			< 0.001^***^
EGC	8	3.29	2.17, 9.72		4.20	3.98, 6.02	
AGC	54	19.70	15.53, 23.57		11.69	7.26, 18.34	
Tumor size (cm)^a^	62			< 0.001^***^			< 0.001^***^
≤ 3	10	4.06	2.55, 12.64		4.24	3.91, 6.06	
> 3	52	19.70	15.23, 23.59		11.97	8.56, 18.35	
Degree of differentiation	62			0.183			0.183
Well	0						
Moderately	16	21.66	11.66, 25.57		12.96	7.03, 23.59	
Poorly	34	17.53	11.75, 21.49		9.18	5.02, 13.15	
N/A	12						
Histologic type	62			0.066			< 0.001^***^
PCC	27	16.7	11.84, 20.42		6.56	4.78, 10.41	
Non-PCC	35	20.21	14.87, 23.89		14.31	9.55, 26.51	
Lauren classification	62			0.161			0.014^*^
Intestinal subtype	20	21.29	10.80, 24.35		11.69	6.96, 17.47	
Non-intestinal subtype	16	16.96	10.58, 20.11		6.47	4.38, 9.52	
N/A	26						

*SUV*_*max*_ maximum standardized uptake value, *IQR* interquartile range, *EGC* early gastric cancer, *AGC* advanced gastric cancer, *PCC* poorly cohesive carcinoma (including signet ring cell carcinoma), *N/A* not applicable

*p* values represent statistical differences in the [^68^Ga]FAPI-04/[^18^F]FDG uptake between subgroups (^*^p < 0.05,^**^p < 0.01,^***^p < 0.001)

^a^Tumor size was analyzed based on PET/CT imaging results

Degree of differentiation, histological type, and Lauren classification were based on known gastroscopy biopsy or postoperative pathology results

### Changes in TNM staging and treatment strategies following [^68^Ga]FAPI-04 and [^18^F]FDG PET/CT

Overall, 57 of 62 patients underwent concurrent CECT for preoperative staging. Supplementary Fig. 1 shows the staging changes following PET/CT scans. In terms of N staging and compared with CECT, two patients were upstaged and one was downstaged by [^18^F]FDG PET/CT, four were upstaged by [^68^Ga]FAPI-04 PET/CT, and five were upstaged by dual-tracer PET/CT. In regards to M staging, five patients were upstaged (including three liver metastases, one lung metastasis, one distant nodal metastasis, and one ovarian metastasis) and five were downstaged (five peritoneal metastases) by [^18^F]FDG PET/CT; five were upstaged (including two liver metastases, two peritoneal metastases, and one distant nodal metastasis) and three were downstaged (including two peritoneal metastases and one distant nodal metastasis) by [^68^Ga]FAPI-04 PET/CT; nine were upstaged (including five liver metastases, one lung metastasis, two peritoneal metastases, one distant nodal metastasis, and one ovarian metastasis) and two were downstaged (including two peritoneal metastases) by dual-tracer PET/CT. Among the above findings, an ovarian metastasis picked up by [^18^F]FDG and dual-tracer PET/CT as well as a liver metastasis picked up by [^68^Ga]FAPI-04 and dual-tracer PET/CT were confirmed to be false-positive uptakes. Additionally, two patients suspected of peritoneal metastases by CECT but negative on PET/CT were proven to have no distant metastases by laparoscopic exploration. The other distant metastases detected in seven patients by dual-tracer PET/CT proved to be true-positive uptakes. Treatment strategies were finally changed in nine patients following [^68^Ga]FAPI-04 and [^18^F]FDG PET/CT scans in accordance with the patients’ clinical conditions and willingness. Seven patients converted from neoadjuvant chemotherapy to conversion therapy. Two patients were excluded from peritoneal metastases (further confirmed by laparoscopic exploration), with one undergoing radical surgery and the other receiving neoadjuvant chemotherapy.

## Discussion

In our study, [^68^Ga]FAPI-04 PET/CT outperformed [^18^F]FDG PET/CT in terms of detecting primary lesions and peritoneal metastases of gastric cancer. However, no statistical difference was observed between the two modalities in detecting nodal metastases. In contrast, [^18^F]FDG PET/CT detected two, two, and one additional liver, lung, and bone metastases, respectively, compared with [^68^Ga]FAPI-04 PET/CT. Furthermore, the dual-tracer PET/CT significantly improved the diagnostic sensitivity of distant metastases compared with either single-tracer PET/CT. Nevertheless, in terms of detecting primary lesions and regional nodal metastases, the dual-tracer PET/CT was not superior to [^68^Ga]FAPI-04 PET/CT. Tumor invasion depth and size were found to be the main factors that affected the avidity of [^68^Ga]FAPI-04 in gastric cancer. Nonetheless, [^68^Ga]FAPI-04 PET/CT showed limited sensitivity in EGC.

In comparison to [^18^F]FDG PET/CT, [^68^Ga]FAPI-04 PET/CT could detect more primary lesions of gastric cancer, with higher SUV_max_ and TBR. Additionally, [^68^Ga]FAPI-04 PET/CT allowed for better detection and visualization of lesion borders, especially in PCC (including SRCC), which [^18^F]FDG PET/CT may easily miss and are consistent with the findings from previous reports [14–18]. Nonetheless, [^68^Ga]FAPI-04 PET/CT demonstrated limited sensitivity in detecting EGC confined to the mucosa and submucosa, i.e., only 37.5% of the primary lesions avid for [^68^Ga]FAPI-04, a sensitivity that was comparable to that of [^18^F]FDG. Our findings revealed that [^68^Ga]FAPI-04 had lower sensitivity (90.3%) in detecting primary lesions of gastric cancer than that in previous reports [14–17]. This may be attributed to the difference in stage distribution and tumor size of the enrolled patients as well as differences among the observers’ interpretations based on visual assessments. Moreover, the sensitivity of dual-tracer PET/CT in detecting primary lesions of gastric cancer was equivalent to that of [^68^Ga]FAPI-04 and higher than that of [^18^F]FDG.

In the diagnosis of regional nodal metastases of gastric cancer, our present patient-based analysis indicated that the sensitivity of [^68^Ga]FAPI-04 PET/CT was not significantly different from that of [^18^F]FDG PET/CT (63.6% vs 54.5%, *p* > 0.05), which was similar to the result of Kuten et al and Jiang et al [14, 16]. However, Pang et al reported a higher sensitivity of [^68^Ga]FAPI-04 than [^18^F]FDG in diagnosing nodal metastases from gastrointestinal tumors (79% vs 54%, *p* < 0.001) [18]. The primary reasons for the limited sensitivity of [^68^Ga]FAPI-04 PET/CT in detecting regional nodal metastases in our study may be attributed to three factors: First, regional and distant nodal metastases were separately analyzed in our study. The diagnosis of regional nodal metastases was based on the postoperative pathology from lymph node dissection, which could potentially increase the number of false-negative lymph nodes compared with distant lymph node analysis. Second, the patients included in the regional lymph node analysis were at a relatively early stage of the disease, and the metastatic lymph nodes might be small and insidious. Additionally, the uptake of small perigastric lymph nodes might be obscured by the radioactive volume effect of the primary gastric tumor and stomach motility. Dual-tracer PET/CT did not significantly improve diagnostic performance in regional nodal metastases compared with either single-tracer PET/CT.

For the detection of distant metastases from gastric cancer, the sensitivity of [^18^F]FDG PET/CT in our study was 73.5%, which was higher than that of the Multicenter Prospective Dutch Cohort Study (PLASTIC) that showed a sensitivity of only 33% [19]. The main reason for this discrepancy would be the different TNM stages of the enrolled patients: the PLASTIC study was restricted to those with locally advanced (≥ cT3 and/or N+, M0) and surgically resectable (< cT4b) gastric cancer after primary staging with CT, whereas advanced patients with distant metastases were also included in our study. Moreover, the lack of follow-up in most patients and a higher proportion of patients with peritoneal metastases in the PLASTIC study also contributed to this discrepancy. As [^18^F]FDG PET/CT was sub-optimal in detecting peritoneal metastases of gastric cancer due to the physiological or inflammatory interference in the intestines and low avidity of [^18^F]FDG in SRCC/MAC [20, 21]. Our present work demonstrated that [^68^Ga]FAPI-04 PET/CT was more sensitive than [^18^F]FDG for detecting peritoneal seeding as it confirmed peritoneal metastases in six additional patients. This superiority was attributed to the lack of physiological accumulation of [^68^Ga]FAPI-04 in the intestines, resulting in a low background uptake in the peritoneal cavity. Additionally, tumor lesions that exceed 2 mm require a supporting stroma, which can be greater in volume than the tumor cells themselves [22]. Therefore, [^68^Ga]FAPI-04 may be more sensitive than [^18^F]FDG even in small lesions, assuming there is sufficient FAP-expressing stroma. Our results were in line with the findings reported by previous studies [15, 17, 23]. However, both [^18^F]FDG and [^68^Ga]FAPI-04 PET/CT missed a small peritoneal metastasis at the top of the diaphragm in one patient, which may be attributed to the spatial resolution restriction of PET and the effect of respiratory movement. Additionally, [^68^Ga]FAPI-04 and [^18^F]FDG PET/CT showed comparable sensitivity in detecting distant nodal metastases, consistent with the results of Qin et al [15]. In the diagnosis of ovarian metastases, [^68^Ga]FAPI-04 detected one additional patient with PCC. However, as a hormone-responsive organ, the physiological uptake of both tracers in the ovaries of premenopausal women may potentially increase the uncertainty in the interpretation of ovarian lesions.

**Fig. 6:**
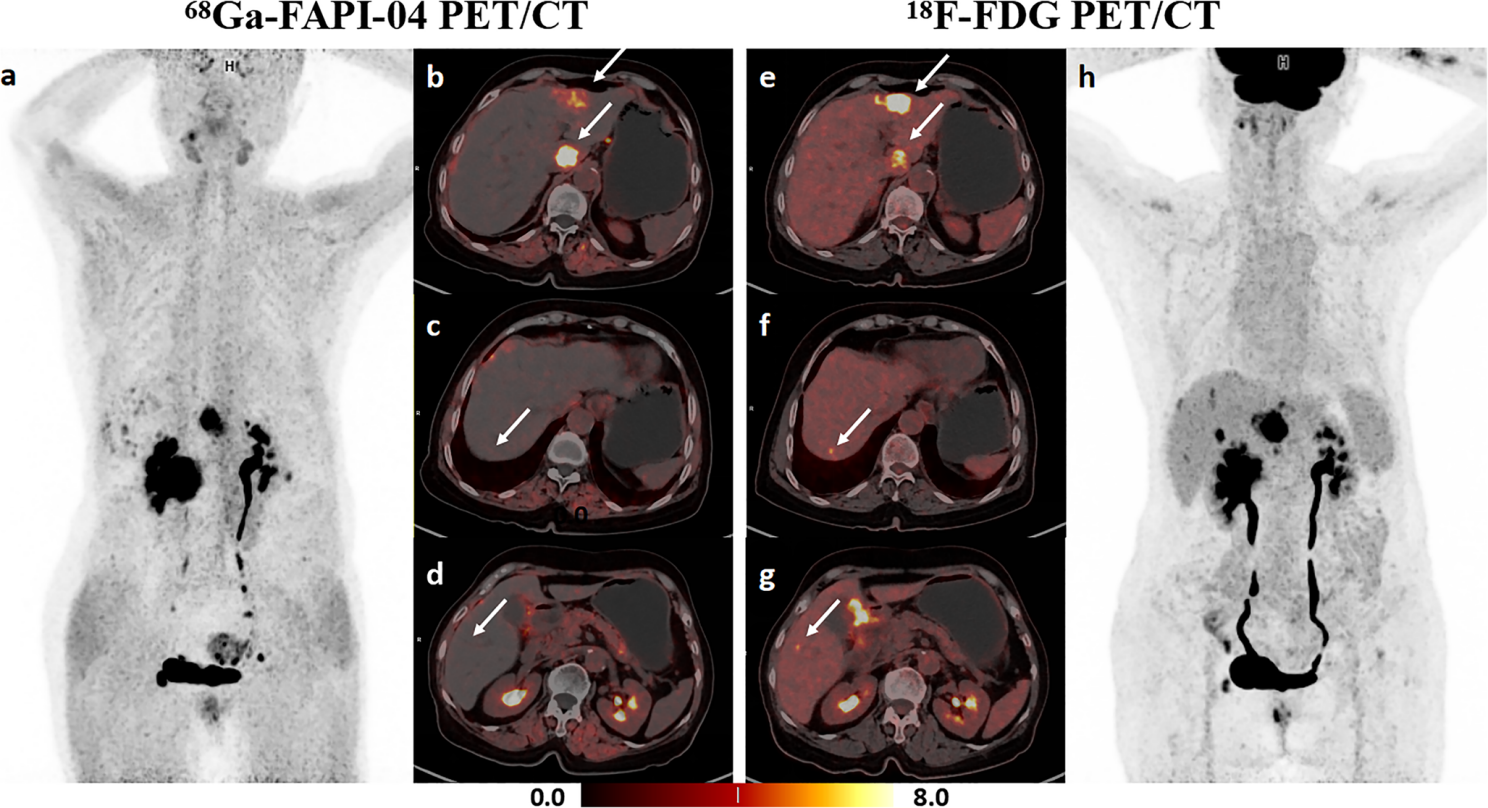
A 71-year-old female patient was histopathologically diagnosed with gastric adenocarcinoma with multiple peritoneal metastases confirmed by laparoscopic exploration and liver metastases confirmed by liver MRI. **a–d** [^68^Ga]FAPI-04 PET/CT imaging. Maximal intensity projection (MIP) image of [^68^Ga]FAPI-04 PET (**a**), clear identification of metastases in the S1 and S2/3 of the liver (**b**), false-negative uptake in the S7 (**c**) and S5 (**d**) of the liver. **e-h** [^18^F]FDG PET/CT imaging. MIP image of [^18^F]FDG PET (**h**), clear evidence of metastases in the S1 and S2/3 of the liver (**e**), but the uptake levels were inconsistent with [^68^Ga]FAPI-04, focal uptake in the S7 (**f**) and S5 (**g**) of the liver

With respect to liver, lung, and bone metastases, [^18^F]FDG PET reportedly performed well, with a sensitivity of 95.2% and a specificity of 100% [24]. ^68^Ga-FAPI PET/CT was found to outperform [^18^F]FDG PET/CT in detecting liver metastases from gastrointestinal cancer [25]. In our research, however, [^18^F]FDG PET/CT recognized three additional liver metastases, which were all missed by [^68^Ga]FAPI-04 PET/CT, whereas one of the liver metastases detected by [^68^Ga]FAPI-04 PET/CT was a false-positive uptake. Figure 6 shows a typical case of our findings. A similar result was obtained by Zhang et al, who found that more liver metastases from pancreatic cancer were detected by [^18^F]FDG PET compared with [^68^Ga]FAPI-04 (*p* < 0.001) [26]. Furthermore, Wang et al reported that ^68^Ga-FAPI PET/CT performed comparably to [^18^F]FDG PET/CT in detecting lung metastases from lung cancer [27]. In our present study, however, [^18^F]FDG PET/CT detected two lung metastases that were missed by [^68^Ga]FAPI-04. Regarding bone metastases, Wu et al found that [^68^Ga]FAPI-04 PET/CT detected more bone metastases from various cancers (100% vs 81.7%, *p* < 0.01) compared with [^18^F]FDG [28]. In the present research, [^68^Ga]FAPI-04 PET/CT missed one and misinterpreted one bone metastasis. Additionally, our site-based analysis revealed that dual-tracer PET/CT markedly improved the sensitivity of detecting distant metastases compared with either single-tracer PET/CT. [^68^Ga]FAPI-04 PET/CT and [^18^F]FDG PET/CT may complement each other for the initial assessment of distant metastases from gastric cancer.

In the subgroup analysis, large tumor size, AGC, intestinal subtype, and non-PCC histological type were predictors of higher avidity of [^18^F]FDG, which is consistent with the results from previous studies [3, 4]. Moreover, our findings suggested that tumor invasion depth and size, rather than the degree of differentiation, histological type, and Lauren classification, were major factors that might influence the avidity of [^68^Ga]FAPI-04 in gastric cancer. Besides, as observed in our study, [^68^Ga]FAPI-04-negative but [^18^F]FDG-positive metastases were usually small, which may be attributed to the fact that desmoplastic reaction, reflected by [^68^Ga]FAPI-04, potentially lags tumorigenesis which is accompanied by altered glucose metabolism, as reflected by [^18^F]FDG [29].

Several limitations exist in the present study. First, in some patients, pathological information such as Lauren classification and degree of differentiation were missing, resulting in a reduced sample size available for analysis. Second, the patients included mainly had AGC; thus, not each suspected metastatic lesion was pathologically verified; the diagnosis of distant metastases depends on our reference standard of comprehensive clinical information. Third, the number of patients with EGC was limited.

In conclusion, our initial study showed that [^68^Ga]FAPI-04 and [^18^F]FDG dual-tracer PET/CT were complementary and improved the sensitivity of detecting pre-treatment distant metastases in gastric cancer, thus helping to improve treatment stratification for gastric patients. Additionally, it should be noted that [^68^Ga]FAPI-04 had limited efficacy in detecting EGC.

## Supplementary information

ESM 1 (DOCX 223 kb)

## Abbreviations

AGC: Advanced gastric cancer
EGC: Early gastric cancer
FAP: Fibroblast activation protein
FAPI: FAP inhibitor
IQR: Interquartile range
MAC: Mucinous adenocarcinomas
MIP: Maximal intensity projection
NPV: Negative predictive value
PCC: Poorly cohesive carcinoma
PPV: Positive predictive value
SRCC: Signet ring cell carcinomas
SUV_max_: Maximum standardized uptake value
SUV_mean_: Mean standardized uptake value
TBR: Tumor-to-background ratio
VOI: Volume of interest

## Declarations

### Guarantor

The scientific guarantor of this publication is Biao Li, MD, Ph.D.

### Statistics and biometry

Prof. Jian Li kindly provided statistical advice for this manuscript.

### Methodology

• prospective

• diagnostic or prognostic study

• performed at one institution
