# Supplementary material for: Utility of [68Ga]FAPI-04 and [18F]FDG dual-tracer PET/CT in the initial evaluation of gastric cancer
Source: Eur Radiol. 2022 Dec 16;33(6):4355–66. doi: 10.1007/s00330-022-09321-1 (PMC10182135; doi:10.1007/s00330-022-09321-1)

**Supplementary Table 1 Methods for confirming distant metastases of gastric cancer**

| Distant metastases (n = 24) | Confirm methods | No. of patients |
| --- | --- | --- |
| Distant lymph nodes (n = 8) | Histopathological finding by percutaneous biopsy | 2 |
|  | Follow-up imaging | 6 |
| Peritoneum (n = 12) | Histopathological finding by surgery | 2 |
|  | Histopathological finding by endoscopic biopsy | 6 |
|  | Laparoscopic exploration | 4 |
| Ovaries (n = 2) | Histopathological finding by surgery | 1 |
|  | Follow-up imaging | 1 |
| Liver (n = 7) | Contemporaneous liver MRI | 5 |
|  | Follow-up imaging | 2 |
| Lung (n = 2) | Follow-up imaging | 2 |
| Bones (n = 3) | Contemporaneous bone scan | 1 |
|  | Follow-up imaging | 2 |

**Supplementary Fig. 1 Changes in TNM staging following ^68^Ga-FAPI-04 and ^18^F-FDG PET/CT (n = 57)**


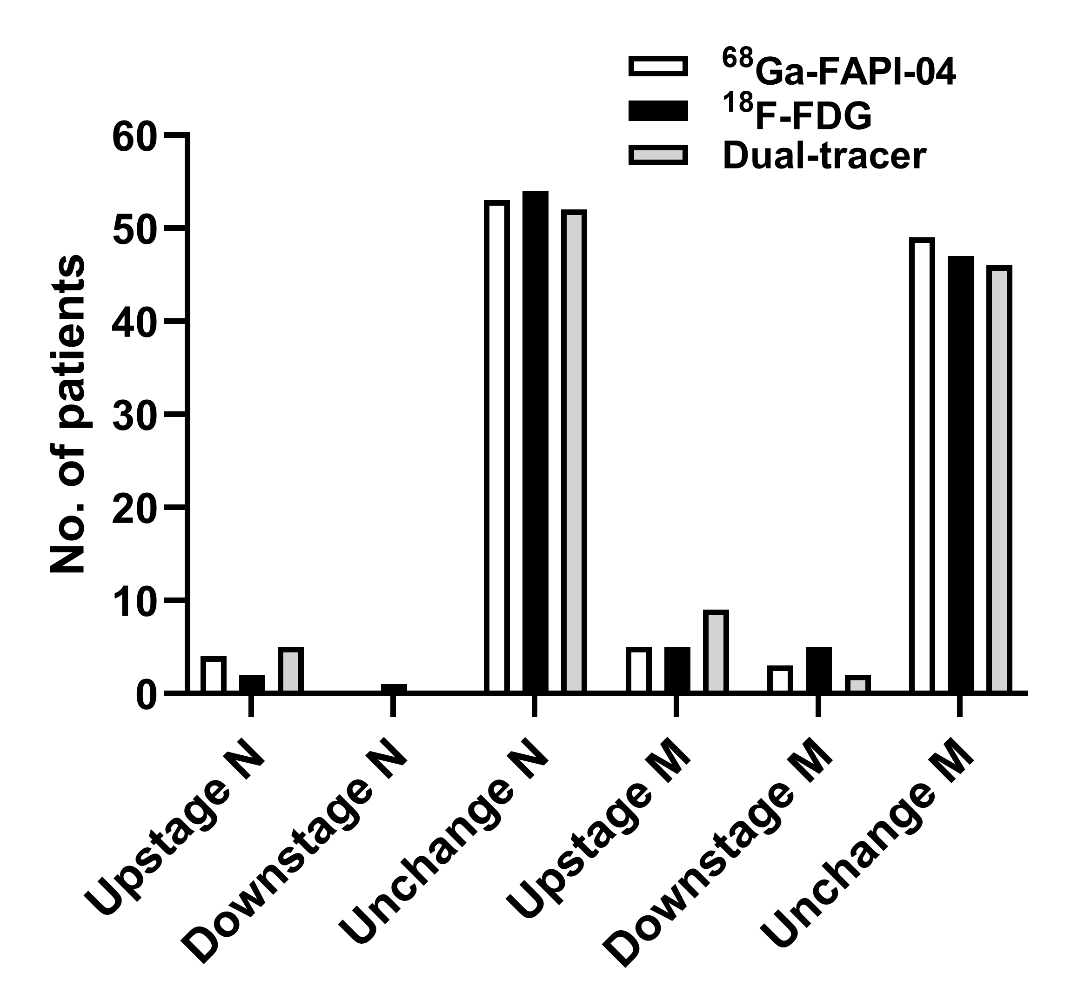

Supplement: Supplementary file 1 — (DOCX 223 kb) [file 330_2022_9321_MOESM1_ESM.docx]
